# Gene expression and prognosis of x-ray repair cross-complementing family members in non-small cell lung cancer

**DOI:** 10.1080/21655979.2021.1964193

**Published:** 2021-09-05

**Authors:** Yongfei Fan, Zhaojia Gao, Xinwei Li, Shuzhang Wei, Kai Yuan

**Affiliations:** a Department of Thoracic Surgery, The Affiliated Changzhou No. 2 People’s Hospital of Nanjing Medical University, Changzhou China; b Heart and Lung Disease Laboratory, the Affiliated Changzhou No. 2 People’s Hospital of Nanjing Medical University, Changzhou China; c Department of Gastroenterology, Affiliated Cancer Hospital of Bengbu Medical College, Bengbu China; d Department of Urology, The Affiliated Changzhou No. 2 People’s Hospital of Nanjing Medical University, Changzhou China

**Keywords:** X-ray repair cross-complementing *(xrcc)* gene family, lung adenocarcinoma (luad), lung squamous carcinoma (lusc), prognosis, bioinformatics

## Abstract

The X-ray repair cross-complementing gene (*XRCC*) family participates in DNA damage repair and its dysregulation is associated with the development and progression of a variety of cancers. However, *XRCCs* have not been systematically studied in non-small cell lung cancer (NSCLC). Using The Cancer Genome Atlas (TCGA) and Oncomine databases, we compared the expression levels of *XRCCs* between NSCLC and normal tissues and performed survival analysis using the data from TCGA. The correlations of *XRCCs* with the clinical parameters were then analyzed using UCSC Xena. Genetic alterations in *XRCCs* in NSCLC and their effects on the prognosis of patients were presented using cBioPortal. SurvivalMeth was used to explore the differentially methylated sites associated with NSCLC and their effect on prognosis. Next, the immunological correlations of *XRCCs* expression level were analyzed using TIMER 2.0. Finally, GeneMANIA was used to visualize and analyze the functionally relevant genes, while Gene Ontology (GO) and Kyoto Encyclopedia of Genes and Genomes (KEGG) were used for functional and pathway enrichment analyses of prognostic genes. Our results revealed that *XRCCs* were overexpressed in lung adenocarcinoma (LUAD) and lung squamous cell carcinoma (LUSC). Univariate and multivariate Cox analyses showed that *XRCC4/5/6* were independent risk factors for LUAD. Additionally, genetic alterations, methylation, and immune cell infiltration demonstrated an association between *XRCC4/5/6* and poor prognosis in LUAD. Finally, the KEGG-enriched and non-homologous end-joining (*NHEJ*) pathways were shown to be associated with *XRCC4/5/6*. In conclusion, our study demonstrated that *XRCC4/5/6* could be used as diagnostic and prognostic biomarkers for LUAD.

## Introduction

Lung cancer remains the leading cause of cancer-related deaths globally, with an estimated diagnosis of 2 million patients and 1.76 million deaths each year[1]. Despite the worldwide efforts to control smoking, which is the most prominent factor causing lung cancer [2,3], the number of patients with lung cancer will only increase further as the use of computed tomography (CT) screening becomes more widespread [4]. Approximately 85% of lung cancer patients are diagnosed with non-small cell lung cancer (NSCLC), with lung adenocarcinoma (LUAD) and lung squamous cell carcinoma (LUSC) being the most common subtypes of NSCLC [5]. Among these two, LUAD is more common and being a peripheral lung cancer, it mostly originates from the bronchial mucosal epithelium, whereas LUSC mainly originates from the bronchial mucosal columnar epithelium and is predominantly a central lung cancer. Although screening high-risk groups using low-dose CT can reduce lung cancer mortality by 20%[6], there is no standard method to predict the survival of patients with NSCLC [7]. Therefore, to develop individualized treatment plans, it is essential to identify prognostic biomarkers and study their oncological characteristics in lung cancer, thereby improving the prognosis of patients with NSCLC.

An underlying hallmark of cancers is their genomic instability, which may be the combined effect of DNA damage, tumor-specific DNA repair defects, and failure to stop or block the cell cycle before the damaged DNA is passed on to the daughter cells[8]. The X-ray repair cross-complementing *(XRCC)* gene family mainly consists of six members *(XRCC1/2/3/4/5/6)*, which are primarily involved in maintaining the chromosome stability by DNA single-strand break repair [9,10], and homologous recombination and non-homologous end-joining to repair the DNA double-stranded breaks [11–14].However, whether the protein kinase, DNA-activated, catalytic subunit (PRKDC), Fanconi anemia (FA) complementation group G (FANCG), breast cancer gene 2 (BRCA2), etc. belong to the XRCC family remains partially controversial. Studies have demonstrated that dysregulation of the XRCC family may disrupt the DNA repair processes, leading to tumor development in the body. [13,15,16]. Despite genomic instabilities promoting the development of cancer, they also offer therapeutic opportunities [17]. Consequently, our research focused on the expression levels and prognostic values of *XRCCs* in NSCLC.

RNA- and DNA-based studies are a significant part of biomedical research, which has been rapidly developing due to the advancements in microarray technologies [18]. Moreover, an increasing number of tumor values are being uncovered due to the improved efficiency of data analysis using the online platforms based on The Cancer Genome Atlas (TCGA) databases, such as UCSC Xena [19], cBioPortal [20], etc. In this study, bioinformatics analysis was used to comprehensively explore the expression and prognosis of XRCC family members in NSCLC and to search for biomarkers that can be used as diagnostic and prognostic markers for NSCLC.

## Materials and Methods

### Acquisition of RNA information

The mRNA data for NSCLC and its subtypes were obtained from TCGA (https://cancergenome.nih.gov/) and Oncomine (https://www.oncomine.org/resource/

login.html) databases in December 2020.

### *Differential expression of* XRCC *family members*

Multiple methods were utilized to determine the expression levels of *XRCC* family members in patients with NSCLC. TCGA database was used to evaluate the differential expression of *XRCC* members between the NSCLC (n = 1037) and normal tissues (n = 108). We then compared the expression levels of *XRCCs* in LUAD (n = 535) and LUSC tissues (n = 502) with those in normal tissues (LUAD, n = 59; LUSC, n = 49). Furthermore, we performed the differential expression analysis of *XRCC* family members in patients with LUAD and LUSC using several datasets from Oncomine.

### *Correlation of the expression levels of* XRCC *family members with clinical parameters in NSCLC*

A correlation analysis of the expression levels of *XRCC* family members with the different clinical stages of cancer (LUAD: stage I, n = 410; stage II, n = 176; stage III, n = 118; stage IV, n = 38; LUSC: stage I, n = 381; stage II, n = 253; stage III, n = 131; stage IV, n = 12) and gender of patients (LUAD: Female, n = 409; Male, n = 343; LUSC: Female, n = 207; Male, n = 559) was performed using the UCSC Xena database (https://xenabrowser.net/). Then, the correlation between the expression levels of *XRCCs* and the gender of patients was analyzed using the Wilcoxon test to identify the significant differences between the two groups, while the correlation between the expression levels of *XRCCs* and the clinical stages of cancer was analyzed using the Kruskal–Wallis test to identify the significant differences among three or more groups.

### Genetic alterations in XRCCs and their prognosis

First, we chose six datasets (n = 2558) (LUAD: TCGA Firehose Legacy; TCGA PanCancer Altas; TCGA Nature 2014; LUSC: TCGA Firehose Legacy; TCGA PanCancer Atlas; TCGA Nature 2014) from cBioPortal (http://www.cbioportal.org/), an open-source website for interactive exploration of multidimensional cancer genomics datasets that aids in the analysis, visualization, and download of a large number of cancer datasets [20,21], to analyze the genetic alterations of *XRCCs* in LUAD and LUSC. Later, we used Kaplan–Meier (KM) analysis to explore the genetic mutations in *XRCCs* and their association with the overall survival (OS) and disease-free survival (DFS) rates of patients. The survival rates were compared by log-rank test to identify the differences between LUAD and LUSC patients with and without the genetic alterations.

### *Prognostic values of* XRCC *family members in LUAD and LUSC*

The prognostic values of the expression levels of *XRCC* family members in LUAD and LUSC were estimated using the data downloaded from TCGA in December 2020 (LUAD: OS, n = 504). The OS rates of LUAD and LUSC patients were analyzed by dividing the patients into low- and high-expression groups based on their median mRNA levels. We then evaluated the differences in the OS rates of the high- and low-expression groups using the KM survival curve. P-value < 0.05 was considered as statistically significant. We further explored the prognosis of *XRCCs* by performing univariate and multivariate Cox analyses to identify the genes that can be considered as independent prognostic factors.

### Analysis of XRCC4/5/6 DNA methylation sites and their prognosis

We performed the differential methylation analysis of *XRCC4/5/6* promoter regions in patients with LUAD using the SurvivalMeth database (http://bio-bigdata.hrbmu.edu.cn/

survivalmeth/) (P < 0.05), a web server to investigate the effects of DNA methylation-related functional elements on prognosis. Additionally, we used the T-test to examine the data. The methylation sites associated with LUAD were classified into high- and low-risk groups for survival analysis using the KM method.

### Tumor purity and immune cell infiltration of XRCC4/5/6

We used TIMER 2.0 (http://timer.cistrome.org/), a comprehensive resource for systematic analysis of immune infiltrates across diverse cancer types [22], to investigate the correlation of expression levels of *XRCC4/5/6* genes with tumor purity and immune cell infiltration in LUAD.

### Genetic interaction analysis

We used the GeneMANIA (http://www.genemania.org), a prediction website that serves as a biological network integrator for gene prioritization and function prediction [23], to construct gene networks associated with *XRCC4/5/6* and visualize the functional correlation between these genes.

### Functional and pathway enrichment analyses

Functional enrichment analysis was performed using Gene Ontology (GO), while pathway enrichment analysis was performed using the Kyoto Encyclopedia of Genes and Genomes (KEGG). Both GO and KEGG were performed using the R package of ‘enrichplot’(v.3.12), which implements several visualization methods to interpret the functional enrichment results obtained from over-representation analysis (ORA) or gene set enrichment analysis (GSEA). Next, we used the KEGG database (https://www.genome.jp/kegg/), a database resource for understanding high-level functions and utilities of the biological system from molecular-level information, especially large-scale molecular datasets generated by genome sequencing and other high-throughput experimental technologies, to plot pathway maps associated with the target genes [24,25].

### Statistical Analysis

Bioinformatics statistical analysis was performed using ‘R x64 4.0.5’ software and open online websites. Differences in expression of *XRCCs* in NSCLC compared with normal tissues were analyzed by Student’s t-test. Genetic alteration prognostic plots and Kaplan-Meier survival curves were compared by logrank test. In all analyses, differences were considered statistically significant if the P value was less than 0.05.

## Results

In this study, we analyzed the expression and prognosis of the *XRCCs* family from multiple biological perspectives using bioinformatics approaches, and we identified that *XRCC4/5/6* may become new biomarkers for LUAD diagnosis and prognosis. This discovery promises to benefit lung cancer patients.

### XRCC *family members are significantly overexpressed in NSCLC*

**Figure 1.**
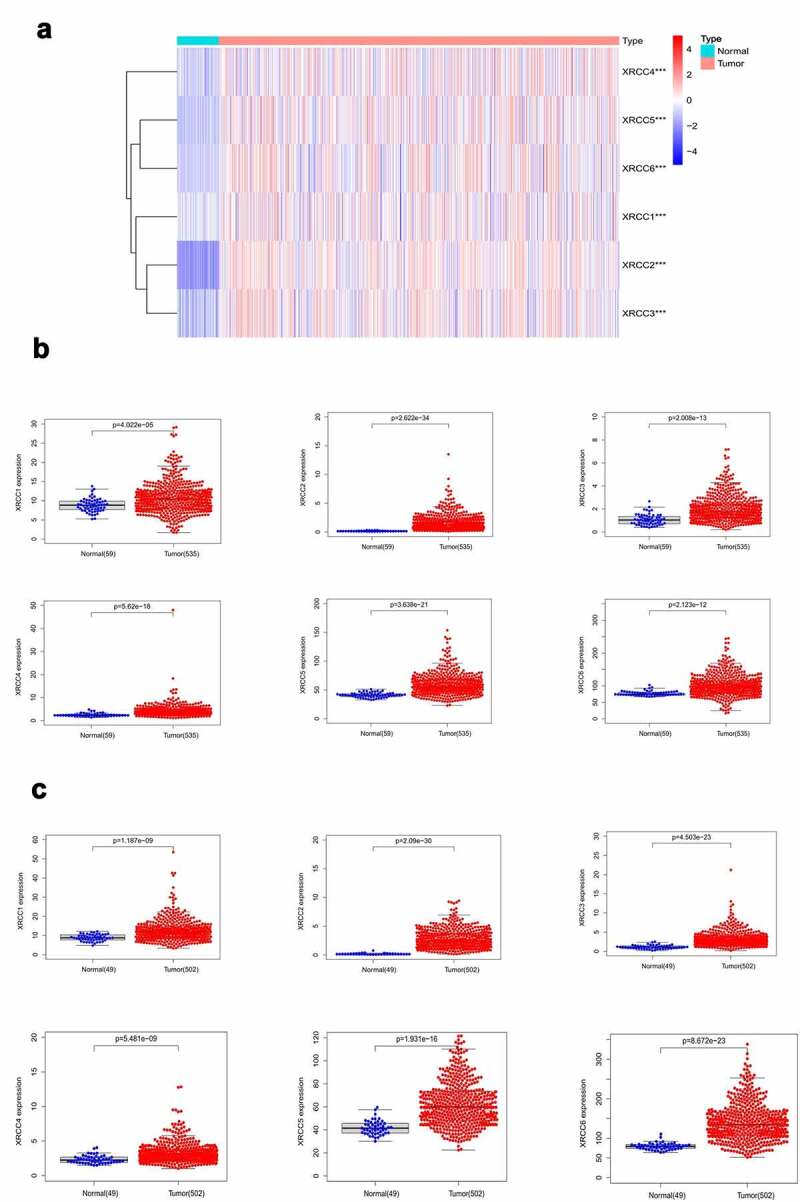
Differential expression analysis of XRCC family members in NSCLC. (A) XRCCs were significantly overexpressed in NSCLC (***P value <0.001).(B) XRCCs were significantly up-regulated in LUAD.(C) XRCCswere significantly up-regulated in LUSC

Using TCGA database, we compared the expression levels of *XRCC* family members between the NSCLC tumor samples (n = 1037) and normal tissue samples (n = 108). The results indicated that *XRCC1/2/3/4/5/6* were expressed at higher levels in NSCLC tissues than in the normal tissues (P < 0.001) (Figure 1(A)). We then analyzed the mRNA levels of *XRCCs* in LUAD and LUSC using TCGA database and found that the expression levels of *XRCC1/2/3/4/5/6* were significantly upregulated in both LUAD and LUSC tissues compared to those in normal tissues (P < 0.05) (Figure 1(B, C)). In addition, we validated these findings by analyzing the datasets of Hou [26], Semalat [27], Su [28], Bhattacharjee [29], Garber [30], Talbot [31], and Yamagata [32] from the Oncomine database (Table 1). Taken together, these results indicate that the expression levels of *XRCC* family members are significantly upregulated in both subtypes of NSCLC (LUAD and LUSC).).

**Table 1.** Significant changes in XRCC family transcription levels between LUAD and LUSC and normal tissues

Gene	Types of lung cancer vs. Normal tissues	Fold change	P value	t-Test	Reference
XRCC1	Lung Adenocarcinoma vs. Normal	1.094	0.014	2.258	Hou[26]
	Squamous Cell Lung Carcinoma vs. Normal	1.223	2.11E-4	3.903	Hou [26]
XRCC2	Lung Adenocarcinoma vs. Normal	3.692	1.84E-10	6.925	Semalat [27]
	Squamous Cell Lung Carcinoma vs. Normal	1.697	1.44E-8	7.107	Hou [26]
XRCC3	Lung Adenocarcinoma vs. Normal	2.008	0.001	3.158	Su [28]
	Squamous Cell Lung Carcinoma vs. Normal	2.787	5.44E-4	3.553	Bhattacharjee [29]
XRCC4	Lung Adenocarcinoma vs. Normal	1.605	4.58E-4	3.511	Su [28]
	Squamous Cell Lung Carcinoma vs. Normal	1.166	0.017	2.216	Hou [26]
XRCC5	Lung Adenocarcinoma vs. Normal	1.879	1.07E-4	6.063	Garber [30]
	Squamous Cell Lung Carcinoma vs. Normal	1.789	9.02E-7	5.448	Talbot [31]
XRCC6	Lung Adenocarcinoma vs. Normal	1.957	0.013	2.795	Yamagata [32]
	Squamous Cell Lung Carcinoma vs. Normal	1.995	0.011	3.616	Yamagata [32]

### *Correlation of the expression levels of* XRCCs *with the clinical parameters of LUAD and LUSC*

**Figure 2.**
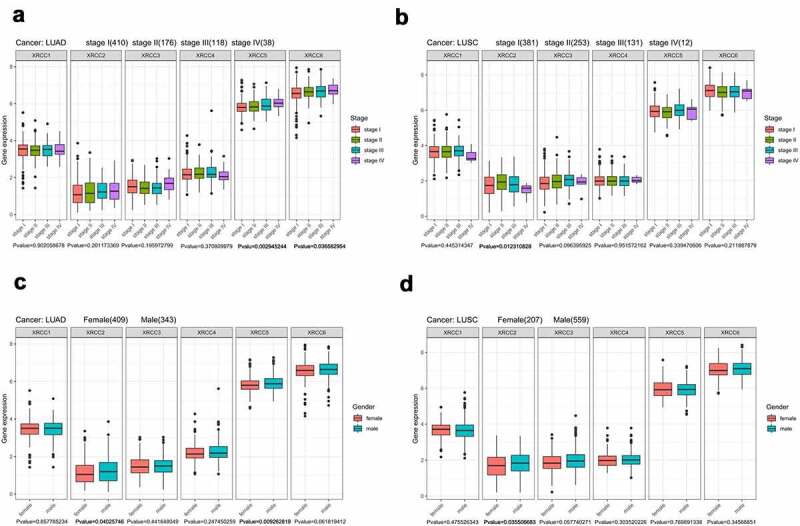
Correlation of XRCC family with clinical factors. (A) Correlation between XRCCs expression and tumor stage in LUAD patients. (B) Correlation between XRCCs expression and tumor stage in LUSC patients. (C)Correlation between XRCCs expression and gender in LUAD patients.(D) Correlation between XRCCs expression and gender in LUSC patients

We used the UCSC Xena database to continue exploring the expression levels of *XRCCs* with regard to the tumor stages and genders of patients with LUAD and LUSC. Statistically significant differences were observed in the patients with LUAD in the *XRCC5* and *XRCC6* groups (P < 0.05), with a positive correlation between the tumor stage and gene expression (Figure 2(A)). In patients with LUSC, *XRCC2* expression was correlated with tumor stage (P < 0.05), with the highest gene expression being observed in stage II (Figure 2(B)). In addition, the mRNA levels of *XRCC2* and *XRCC5* in LUAD patients and *XRCC2* in LUSC patients were higher in men than in women (P < 0.05; Figure 2(C, D)). Overall, these findings imply that the expression levels of *XRCCs* are partially correlated with the clinical parameters in NSCLC patients.

### Genetic alterations in XRCCs and their prognostic significance

**Figure 3.**
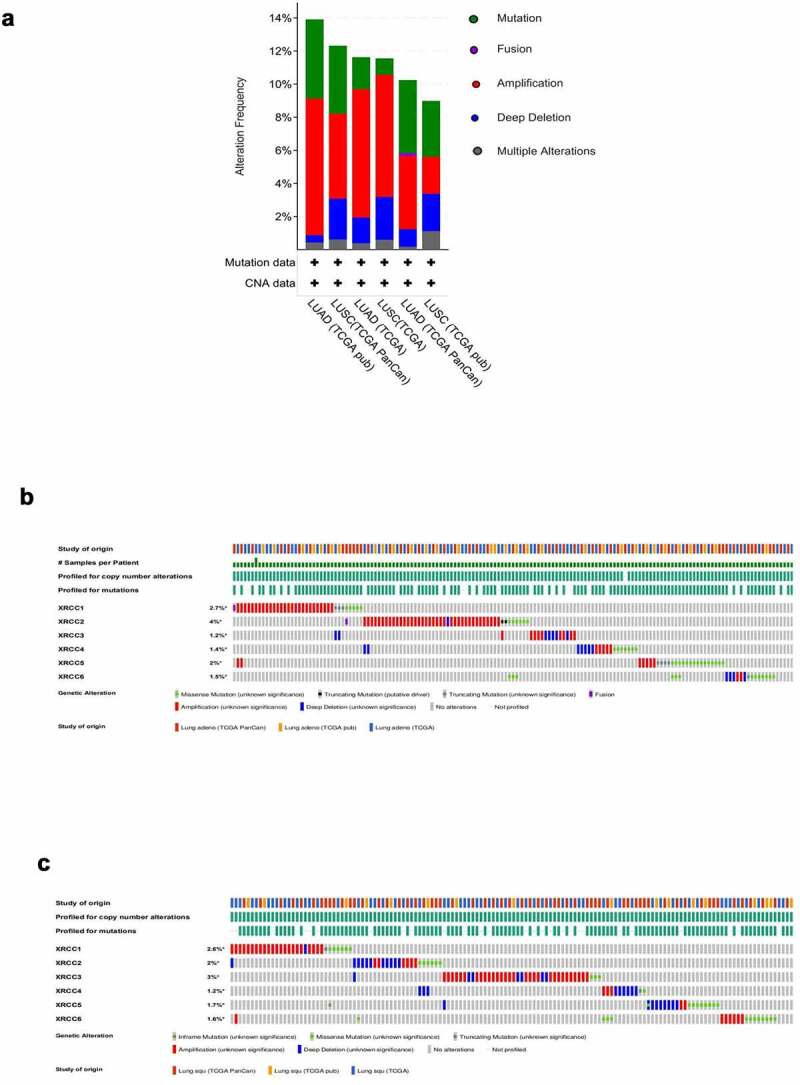
Genetic alterations in XRCC family in patients with LUAD and LUSC. (A)The genetic alterations of XRCCs in six datasets based on TCGA.(Lung Adenocarcinoma– TCGA Firehose Legcay ;TCGA PanCancer Altas ;TCGA Nature 2014; Lung Squamous Carcinoma – TCGA Firehose Legcay ;TCGA PanCancer Altas ;TCGA Nature 2014) (B).XRCCs alternations in LUAD. (C).XRCCs alternations in LUSC

Six TCGA-based datasets were selected from the cBioPortal online tool to analyze the genetic alterations in the *XRCC* family members in patients with LUAD and LUSC. Among the analyzed datasets, the frequency of genetic alterations in LUAD and LUSC, including amplifications, mutations, fusions, deep deletions, and multiple alterations, ranged from 8.99% (16/178) to 13.91% (32/230). Amplification was the most common alteration observed in the six datasets, while mutation and deep deletion ranked second and third, respectively (Figure 3(A)). The percentages of genetic alterations in *XRCCs* ranged from 1.2% to 4% in patients with LUAD (*XRCC1*, 2.7%; *XRCC2*, 4%; *XRCC3*, 1.2%; *XRCC4*, 1.4%; *XRCC5*, 2%; *XRCC6*, 1.5%) (Figure 3(B)), and from 1.2% to 3% in patients with LUSC (*XRCC1*, 2.6%; *XRCC2*, 2%; *XRCC3*, 3%; *XRCC4*, 1.2%; *XRCC5*, 1.7%; *XRCC6*, 1.6%) (Figure 3(C)).

**Figure 4.**
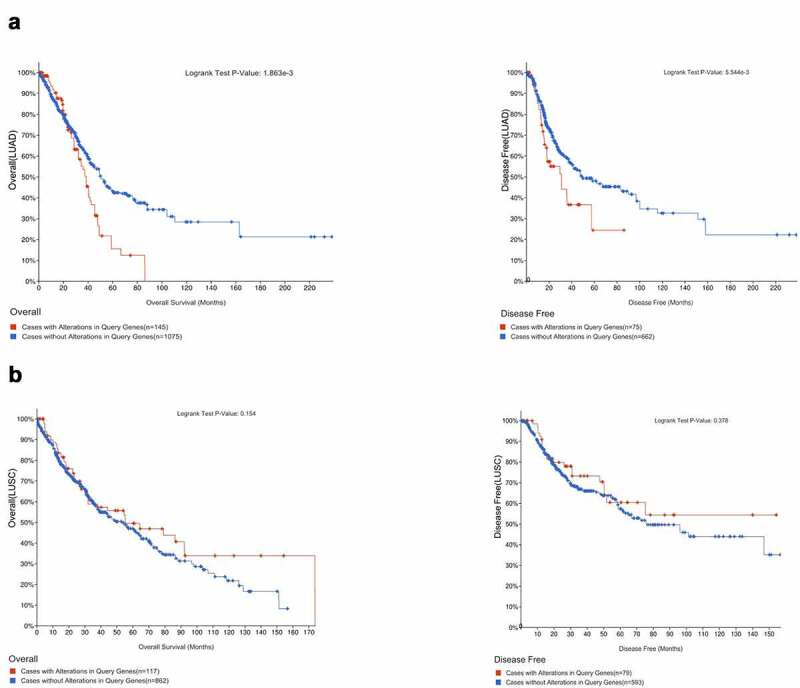
Correlation between the genetic alterations of XRCCs and prognosis of patients with LUAD and LUSC.(A) Correlation of genetic alterations in XRCCs with OS and DFS in LUAD patients.(B) Correlation of genetic alterations in XRCCs with OS and DFS in LUSC patients

Next, we explored the relationship between these genetic alterations in *XRCCs* and the survival rates of patients with LUAD and LUSC. KM analysis showed that the presence of altered *XRCC* family members was associated with reduced OS and DFS in LUAD patients compared to that in patients with unaltered *XRCC* family members (P < 0.05) (Figure 4(A)). In contrast, the analysis of genetic alterations in *XRCCs* in patients with LUSC did not reveal any significant correlation with the OS and DFS (Figure 4(B)). Nevertheless, the curve trend showed that LUSC patients with altered *XRCCs* were predicted to exhibit better OS than those with unaltered (Figure 4(B)). In summary, these results suggest that genetic alterations in *XRCC* family members significantly affect the prognosis of patients with LUAD and LUSC.

### *Prognostic values of* XRCC *family members in LUAD and LUSC*

**Figure 5.**
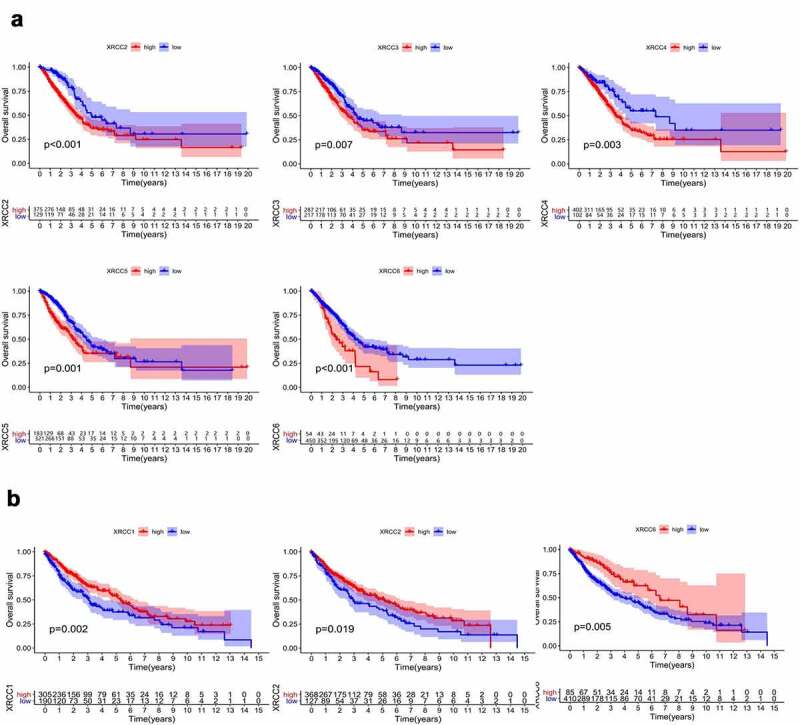
Prognostic value of XRCC family mRNA expression in LUAD and LUSC. (A)The expression of XRCCs in LUAD associated with OS. (B)The expression of XRCCs in LUSC associated with OS

We analyzed the prognostic values of *XRCCs* in patients with LUAD and LUSC using TCGA database. The results suggested that high expression levels of *XRCC2/3/4/5/6* were significantly associated with poor OS in patients with LUAD patients (Figure 5(A)). Additionally, upregulation of the mRNA levels of *XRCC1/2/6* was significantly associated with longer OS in patients with LUSC (Figure 5(B)). These results are consistent with those shown in Figures 4A and 4B.

**Figure 6.**
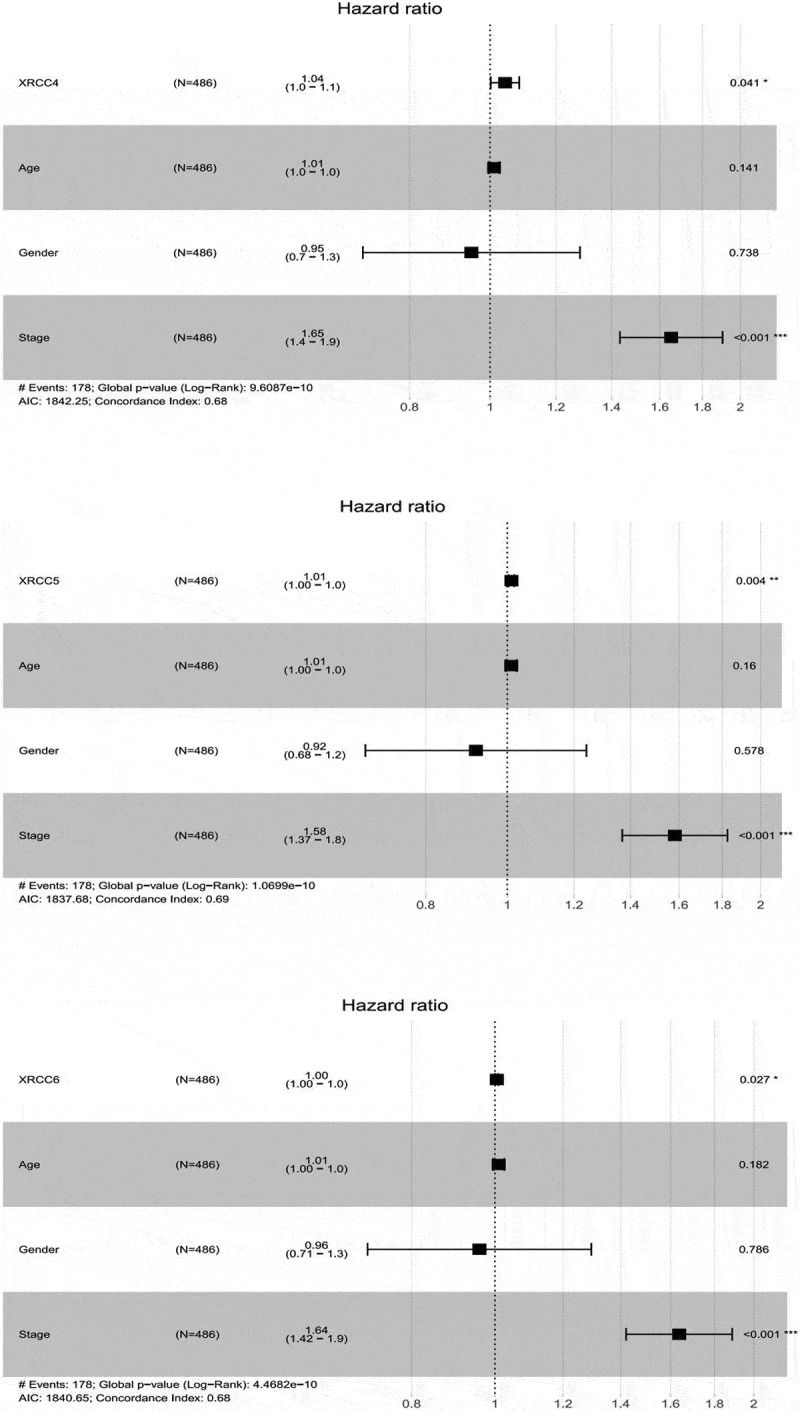
Multifactorial Cox analysis for independent prognostic analysis

Subsequently, we used univariate and multivariate Cox analyses by combining the target genes, age, sex, and staging parameters to identify genes that can be used as prognostic indicators independent of these clinical factors. Univariate Cox analysis showed that the tumor stage, *XRCC4, XRCC5*, and XRCC6 were potential risk factors for the OS in patients with LUAD, while no such statistically significant genes were found in patients with LUSC (P < 0.05) (Table 2; Table 3). In addition, multivariate Cox analysis demonstrated that the tumor stage, *XRCC4, XRCC5*, and *XRCC6* could predict the tumor prognosis independent of other factors for the OS in patients with LUAD (P < 0.05; Figure 6). Taken together, our results illustrated the excellent prognostic characteristics of *XRCC* family members in NSCLC patients and also showed that *XRCC4/5/6* were potential independent risk factors for OS in patients with LUAD.

**Table 2.** Univariate analysis of the correlation of XRCCS expression and clinical characteristics with OS among LUAD patients

Parameter	Univariate analysis		
	HR	95%CI	*P*-value
XRCC1	1.012	0.9833~1.041	0.4165
XRCC2	1.059	0.9585~1.171	0.2583
XRCC3	1.019	0.9037~1.149	0.7589
XRCC4	1.047	1.003~1.092	**0.03644**
XRCC5	1.016	1.008~1.023	**2.47E-05**
XRCC6	1.005	1.001~1.010	**0.008255**
Age	1.008	0.9927~1.023	0.3120
Gender	1.102	0.8208~1.480	0.5178
Stage	1.632	1.420~1.876	**5.32E-12**

**Table 3.** Univariate analysis of the correlation of XRCCS expression and clinical characteristics with OS among LUSC patients

Parameter	Univariate analysis		
	HR	95%CI	*P*-value
XRCC1	0.9876	0.9611~1.015	0.3711
XRCC2	0.9884	0.9081~1.076	0.7864
XRCC3	0.9435	0.8745~1.018	0.1334
XRCC4	1.020	0.9330~1.116	0.6596
XRCC5	1.000	0.9940~1.006	0.9780
XRCC6	0.9984	0.9956~1.001	0.2502
Age	1.017	1.000~1.034	**0.0444**
Gender	1.196	0.8675 ~1.650	0.2743
Stage	1.256	1.064~1.482	**0.007026**

### *Correlation of methylation of* XRCC4/5/6 *with survival analysis in LUAD*

**Figure7.**
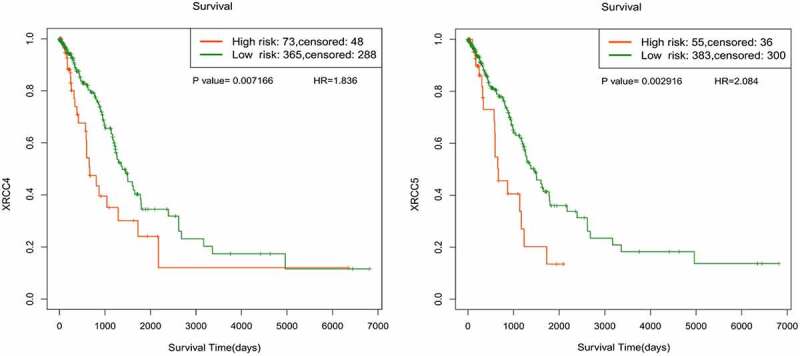
Survival analysis of XRCC4/5/6 methylation sites associated with LUAD

Based on our finding that *XRCC4/5/6* can be used as potential independent risk factors for patients with LUAD, we further analyzed the methylation sites of *XRCC4/5/6* using SurvivalMeth. Comparison of the methylation levels in LUAD tumor tissues with those in normal tissues identified three relevant methylation sites each in *XRCC4* and *XRCC5* (P < 0.05), whereas no such methylation sites were found in *XRCC6* (P < 0.05) (Table 4). Then, we divided the samples with differentially methylated sites into high- and low-risk groups and performed survival analysis using the KM method. The results showed that the *XRCC4/5* high-risk groups were associated with a poor prognosis (Figure 7), which is consistent with the conclusions of our previous survival analysis.

**Table 4.** Analysis of XRCC4/5/6 methylation site differences in LUAD and normal tissues

Gene	Site	Average of tumor	Average of normal	Delta value	P-value
XRCC4	cg20536432	0.7958	0.8501	-0.05430	4.63e-09
	cg09507411	0.7662	0.7400	0.02613	0.00478
	cg15150652	0.8391	0.8275	0.01152	0.04637
XRCC5	cg00723275	0.8132	0.7941	0.01907	0.004026
	cg09977847	0.6640	0.7173	-0.05331	5.633e-07
	cg13506354	0.8671	0.8531	0.01401	0.01698

### *Correlation analysis of expression levels of* XRCC4/5/6 *and immune cell infiltration*

**Figure8.**
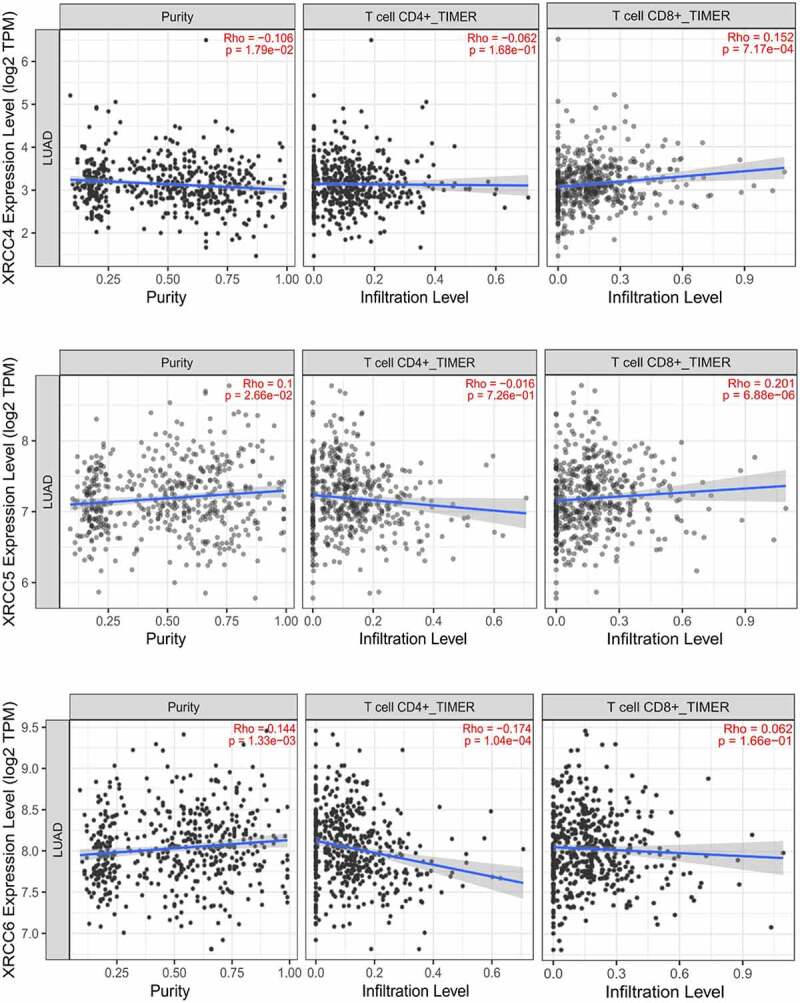
Tumor purity and immune cell infiltration associated with XRCC4/5/6 expression in patients with LUAD

Using TIMER 2.0, we analyzed the correlation between the expression levels of *XRCC4/5/6* and the level of immune cell infiltration in patients with LUAD. Our results indicated that the expression levels of *XRCC5/6* were positively correlated, while those of *XRCC4* were negatively correlated with tumor purity (Figure 8). High expression levels of *XRCC4/5/6* showed a significant negative correlation with the infiltration of the cluster of differentiation 4 (CD4)^+^ T cells and positive correlation with the infiltration of the cluster of differentiation 8 (CD8)^+^ T cells (Figure 8). The immune cell composition of NSCLC tissues was dominated by T cells (47%), with CD4^+^ T cells being the most abundant T cell population (26%), followed closely by CD8^+^ T cells (22%) [33]. Furthermore, a study demonstrated that low-infiltrating CD4^+^ T cells, high-infiltrating CD8^+^ T cells, and high-infiltrating CD8^+^/low-infiltrating CD4^+^ T cells were associated with a poor prognosis in patients with NSCLC [34,35]. Taken together, our research showed that the infiltration of CD4 + T and CD8 + T cells associated with expression levels of *XRCC4/5/6* revealed poor prognosis.

### *Gene-function interaction analysis of* XRCC4/5/6

**Figure 9.**
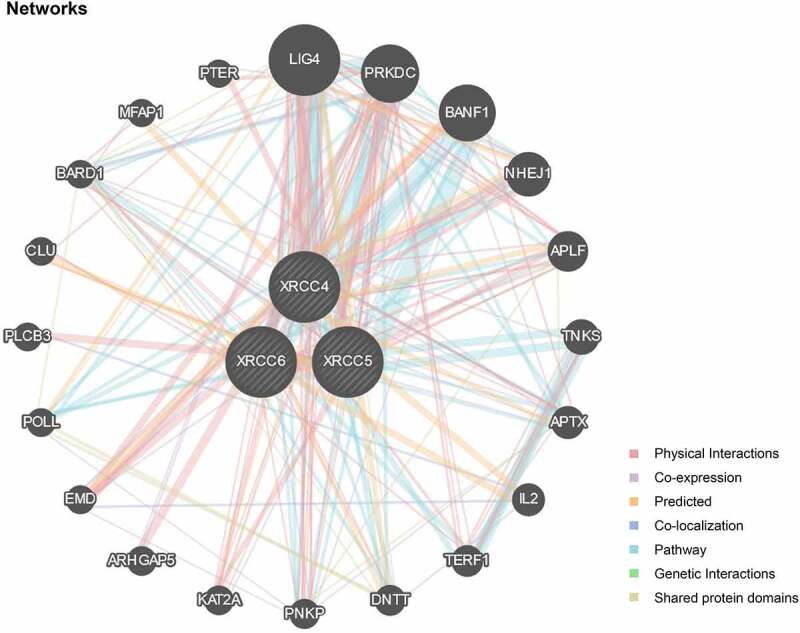
Network of gene interactions associated with XRCC4/5/6 function

To identify the genes associated with *XRCC4/5/6* functions, we performed a visual analysis using the GeneMANIA online tool and identified 20 genes that closely interacted with *XRCC4/5/6* (Figure 9). Among them, DNA ligase IV (*LIG4), PRKDC*, barrier-to-autointegration factor 1 (*BANF1*), non-homologous end-joining gene 1 (*NHEJ1*), and aprataxin and polynucleotide kinase 3ʹ-phosphatase (*PNKP*)-like factor (*APLF*) were the five most significantly interacting genes with *XRCC4/5/6* (Table 5). *XRCC4/5/6* all had multiple interactions with *LIG4* and *PRKDC*, and the most significant interactions were physical interactions and pathway. However, *BANF1* only interacted with XRCC4/5, and the most relevant interaction was pathway (Figure 9). Therefore, identifying genes that are functionally similar to *XRCC4/5/6* will aid in the discovery of other DNA repair target genes, thereby expanding the therapeutic options for tumor treatment..

**Table 5.** The five most significant genes interacting with XRCC4/5/6

Gene	Description	Rank
LIG4	DNA ligase 4 [Source:HGNC Symbol;Acc:HGNC:6601]	1
PRKDC	protein kinase, DNA-activated,catalytic polypeptide [Source:HGNC Symbol;Acc:HGNC:9413]	2
BANF1	barrier to autointegration factor 1 [Source:HGNC Symbol;Acc:HGNC: 17397]	3
NHEJ1	non-homologous end joining factor 1 [Source:HGNC Symbol;Acc:HGNC: 25737]	4
APLF	aprataxin and PNKP like factor [Source:HGNC Symbol;Acc:HGNC:28724]	5

### Predicting the function and pathway of XRCC4/5/6 in LUAD

**Figure 10.**
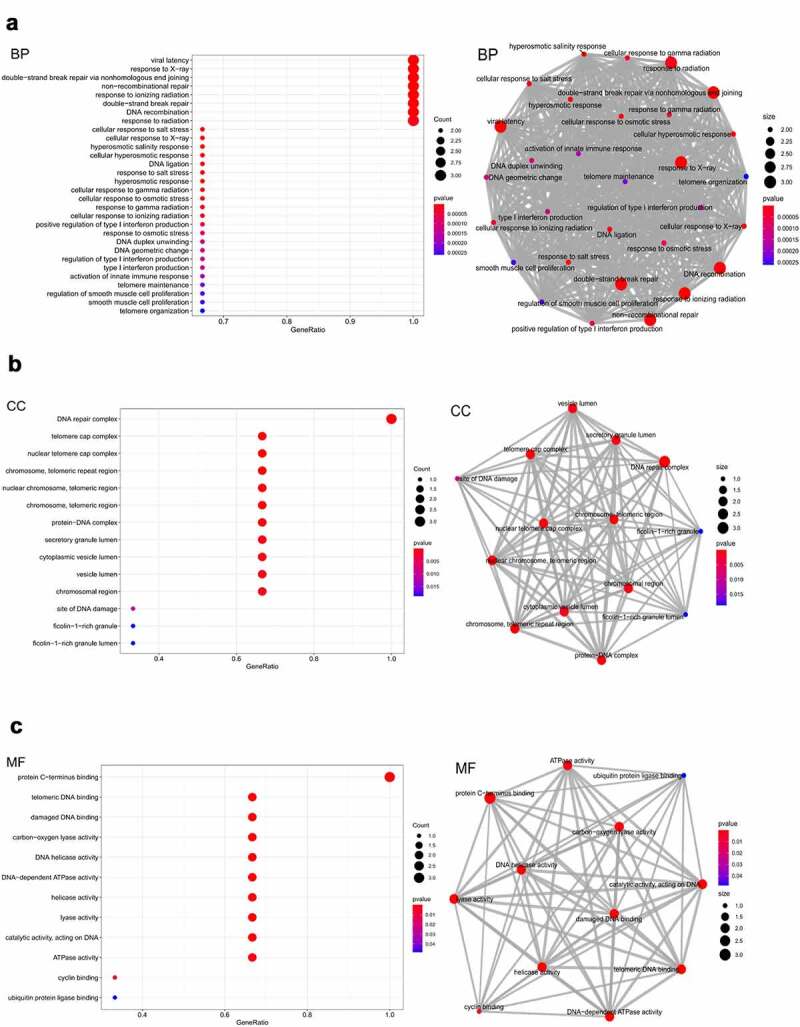
Enrichment of functions and relationships among these functions for XRCC4/5/6 by analyzing GO.(A) Enrichment of functions and relationships among these functions in BP.(B) Enrichment of functions and relationships among these functions in CC.(C) Enrichment of functions and relationships among these functions in MF

The functions of *XRCC4/5/6* and the correlations among their functions were explored by GO enrichment analysis in terms of biological processes (BP), cellular components (CC), and molecular functions (MF) [36]. The results showed that the functions significantly regulated by *XRCC4/5/6* concerning BP were viral latency, response to x-rays, double-strand break repairs via non-homologous end-joining, non-recombinational repair, response to ionizing radiations, double-strand break repair, DNA recombination, and response to radiation (Figure 10(A)). Moreover, these eight functions had the most significant functional correlation (Figure 10(A)). For CC, the function of the DNA repair complex was most markedly controlled by *XRCC4/5/6* alterations and was most significantly correlated with other regulated functions (Figure 10(B)). Among all the MFs, the function most significantly regulated by *XRCC4/5/6* was the protein C-terminal-binding, which was most significantly correlated with other regulatory functions (Figure 10(C)).

**Figure 11.**
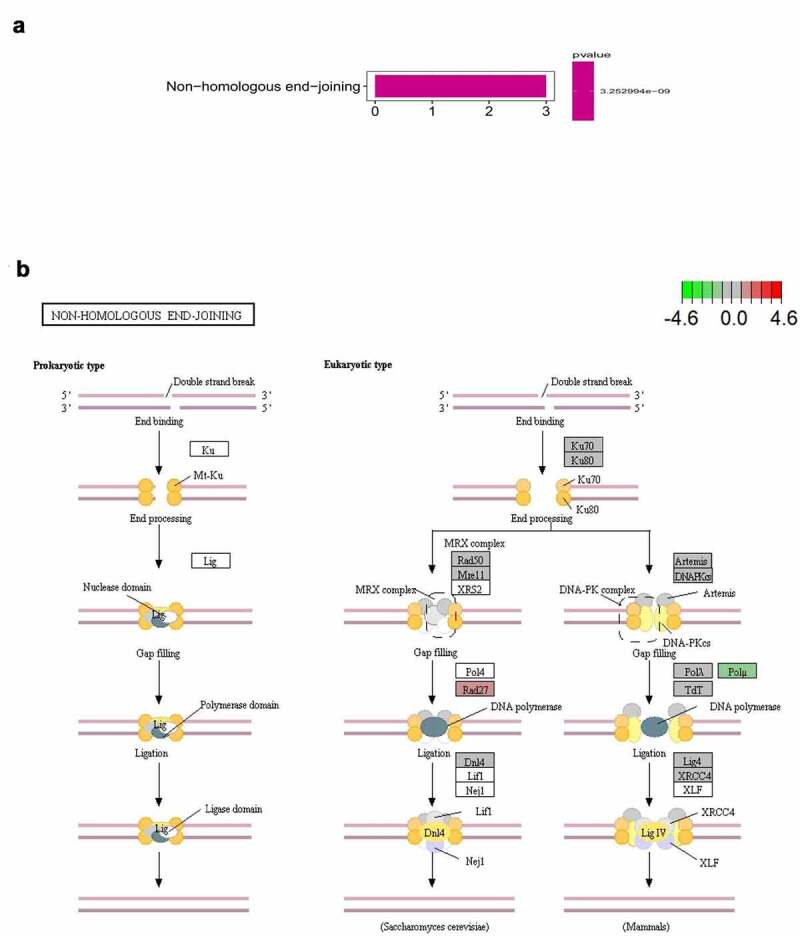
Pathway predicted for XRCC4/5/6 in LUAD.(A) Identify relevant pathways by KEGG enrichment analysis.(B) Mapping relevant pathways through the KEGG website

KEGG enrichment analysis was used to identify pathways related to the functions of *XRCC4/5/6* alterations and the pathways of gene alterations were drawn using the KEGG website. However, by KEGG enrichment analysis, only the *NHEJ* pathway was found to be associated with *XRCC4/5*/*6* alterations in LUAD (P < 0.05) (Figure 11(A)). Furthermore, we mapped the *NHEJ* pathway using the KEGG website, which can directly reflect the changes in genes in the pathway. The results showed that Rad27 was highly expressed in the *Saccharomyces cerevisiae* pathway, while DNA polymerase μ (Polμ) was expressed at low levels in the mammalian pathway (Figure 11(B)).

## Discussion

Environmental factors play critical roles in tumorigenesis by affecting the stability of target genes [37]. Therefore, the ability of the genes to repair the damaged DNA is closely related to tumorigenesis and determines the difference in the susceptibility to cancer in different individuals [38]. Although the *XRCC* family members are essential components of DNA repair mechanisms, they are reported to play potential roles in the tumorigenesis and prognosis of multiple cancers; however, their oncological and prognostic values in NSCLC need to be investigated in future studies.

*XRCC1*, a single-strand break repair factor [39], has been reported to play essential roles in various cancers. Miao et al. demonstrated that the *XRCC1* Arg399Gln polymorphism is a genetic susceptibility factor for the development of gastric cardia cancer [40]. The *XRCC1 399Gln* allele is a potentially important determinant of susceptibility to smoking-induced pancreatic cancer [41]. Moreover, the presence of single nucleotide polymorphism (SNP)-77 T > C in the 5′-untranslated region (UTR) of *XRCC1* is associated with an increased risk of developing lung cancer [42]. In our study, *XRCC1* was highly expressed in both LUAD and LUSC tissues. Moreover, high expression of *XRCC1* in LUSC was associated with longer OS, but it was not statistically significant in LUAD. The frequency of *XRCC1* alterations was 2.6% in LUAD and 2.7% in LUSC, and both of these gene amplifications account for a significant part.

A variety of cancers have been associated with *XRCC2*, such as breast [43], lung [44], pancreatic [45], and head and neck cancers [46]. Zienolddiny et al. reported that there are associations between a set of genetic polymorphisms in DNA repair genes and the risk of lung cancer [44]. Furthermore, in vivo studies using a viral vector containing *XRCC2* promoter indicated that *XRCC2* is a promising target for the diagnosis and treatment of various types of cancers [47]. In this study, *XRCC2* was found to be overexpressed in the LUAD and LUSC tissues compared with the normal tissues. Moreover, high *XRCC2* expression was significantly associated with poor OS in LUAD, while it was associated with better OS in LUSC. In terms of clinical characteristics, the expression of this gene was correlated with the clinical stage of LUSC and the gender of patients with LUAD and LUSC. Moreover, *XRCC2* was predominantly amplified in LUAD exhibiting the highest frequency (4%), while it was predominantly deleted in LUSC (2%).

*XRCC3* is required to repair double-strand breaks via homologous recombination repair pathways for accurate chromosomal segregation and repair of DNA cross links [11,48]. *XRCC3* IVS6 C1571T and the associated haplotype AAC are associated with a relatively high risk of lung cancer [14]. In the current study, *XRCC3* expression was higher in the LUAD and LUSC tissues than in the normal tissues. In terms of survival, high *XRCC3* expression in LUAD was significantly associated with the poor OS of patients, while no such relation was observed in LUSC. In addition, *XRCC3* was the most frequently altered gene of the *XRCC* family in LUAD (3%) and amplification was the dominant genetic alteration in both LUAD and LUSC.

*XRCC4/5/6* are essential components of the *NHEJ* pathway, in which *XRCC4* acts in conjunction with the Ku70-Ku80 heterodimer encoded by *XRCC5* and *XRCC6*, respectively, and ligase IV for precise end-joining of blunt DNA double-strand breaks [12,13,49]. Moreover, *NHEJ* is the primary pathway for the repair of double-strand breaks in mammalian cells [13]. These conclusions are consistent with our pathway enrichment analysis results shown in Figures 11A and 11B. Our study also indicated that Rad27 was highly expressed in the *Saccharomyces cerevisiae* pathway, while Polμ was downregulated in the mammalian pathway. The association between the polymorphisms of genes in the *NHEJ* pathway and the susceptibility and prognosis of lung cancer was first proposed by Tseng et al [50]. Furthermore, *XRCC4/5/6* have been widely studied by scholars in the field of oncology [51–55].

In our study, *XRCC4/5/6* were overexpressed in LUAD and LUSC tissues as compared to those in the normal tissues, which is consistent with the results of Ye et al [56] and Ma et al [57] who reported that *XRCC5* was overexpressed in LUAD and LUSC. Survival analysis showed that high expression levels of *XRCC4/5/6* were associated with poor OS in LUAD patients. Multivariate Cox analysis showed that *XRCC4/5/6* were potential independent risk factors for LUAD. Moreover, *XRCC4/5/6* DNA methylation and immune cell infiltration analysis in LUAD also indicated a poor prognosis. Currently, biomarkers are used for the clinical diagnosis and prognosis in a variety of cancers, such as lung [58], breast [59,60], and gastric cancers [61,62] Therefore, *XRCC4/5/6* can be used as novel diagnostic and prognostic biomarkers for LUAD.

Notably, this study has some limitations. First, the conclusions lack experimental validation; however, we applied a multiple-omics approach and used multiple databases to further support our conclusions. Second, the data in this study are mainly obtained from publicly available databases; therefore, the results heavily depend on the quality of the data in the databases.

## Conclusion

In summary, we comprehensively analyzed the role of *XRCC* family members in NSCLC from multiple oncological perspectives using various online authoritative databases. Our study demonstrated that *XRCC4/5/6* could be used as diagnostic and prognostic biomarkers for LUAD.

## Data Availability

This study analyzed publicly available online datasets. TCGA:https://cancergenome. nih.gov/;UCSC Xena:https://xenabrowser.net/;Oncomine: https://www.oncomine. org/resource/login.html;cBioPortal:http://www.cbioportal.org/;SuvivalMeth:http://bio-bigdata.hrbmu.edu.cn/survivalmeth/; GeneMANIA:http://www.genemania.org;TIMER2.0: http://timer.cistrome.org/; CellMiner: https://discover.nci.nih.gov/cellminer/home.do

## Abbreviations

*XRCC*: X-ray repair cross-complementing; NSCLC: non-small cell lung cancer; LUAD: lung adenocarcinoma; LUSC: lung squamous carcinoma; TCGA: the Cancer Genome Atlas; GO: Gene Ontology; KEGG: Kyoto Encyclopedia of Genes and Genomes; BP: Biological Process; NHEJ: the Non-homologous end-joining; TIMER: the Tumor Immune Estimation Resource; UCSC: the University of California Santa Cruz; OS: Overall Survival; DFS: Disease-Free Survival; BP: biological processes; CC: cellular components; MF: molecular functions
